# Supplementary material for: External Stimuli-Responsive Characteristics of Poly(N,N′-diethylacrylamide) Hydrogels: Effect of Double Network Structure
Source: Gels. 2022 Sep 15;8(9):586. doi: 10.3390/gels8090586 (PMC9498466; doi:10.3390/gels8090586)
Supplement: Supplementary file 1 [file gels-08-00586-s001.zip › gels-1899629-supplementary.pdf]

gels

Supplementary Materials

External Stimuli-Responsive Characteristics of Poly(*N,N′*-diethylacrylamide) Hydrogels: Effect of Double Network Structure

Julie Šťastná, Vladislav Ivaniuzhenkov and Lenka Hanyková *

Department of Macromolecular Physics, Faculty of Mathematics and Physics, Charles University, V Holešovičkách 2, 180 00 Prague 8, Czech Republic
* Correspondence: lenka.hanykova@mff.cuni.cz

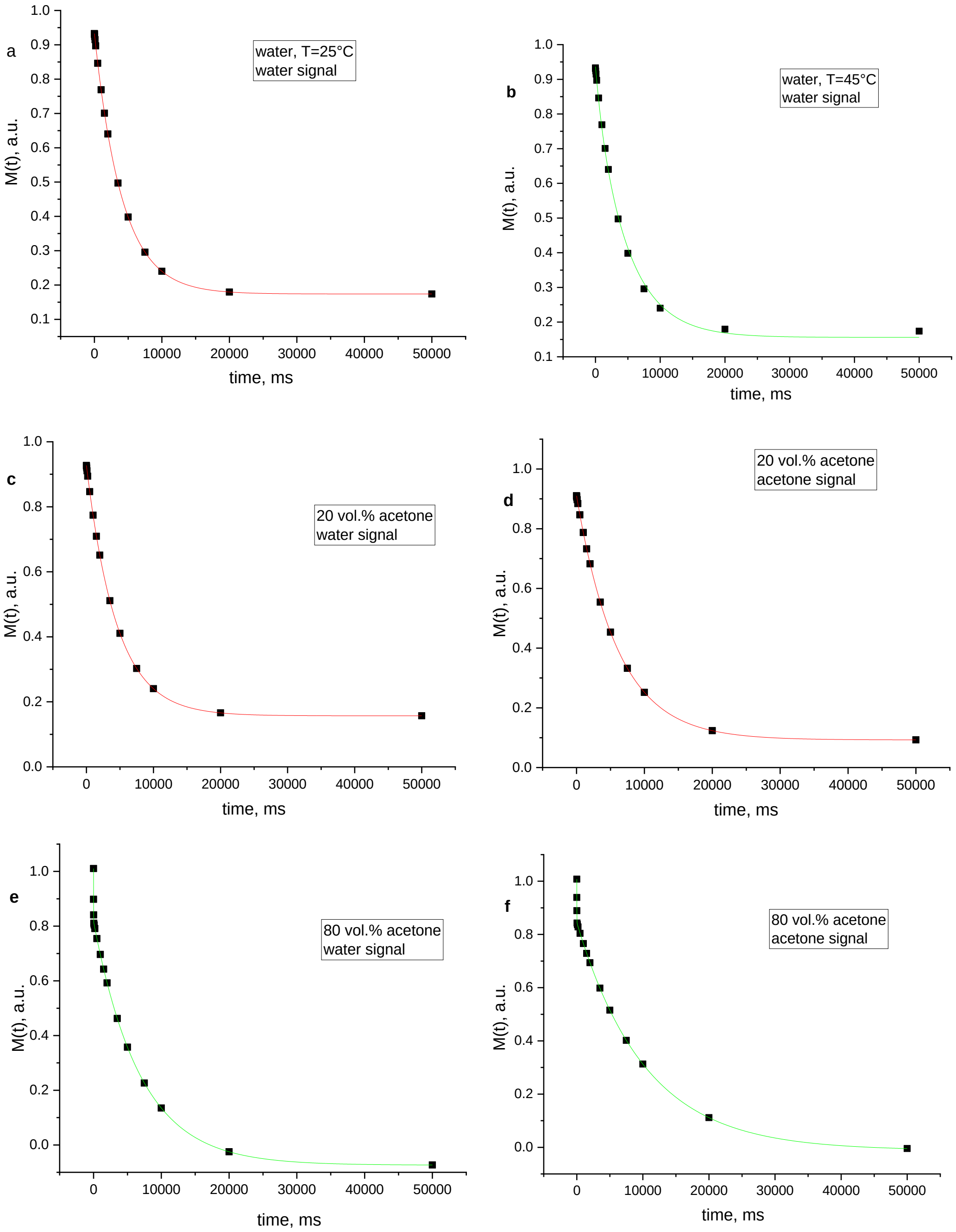

Figure S1. Proton T_2 relaxation curves of solvent molecules in various solutions with DN-DA hydrogel: (**a**) water, T=25°C, (**b**) water, T=45°C, (**c**, **d**) 20 vol.% acetone, (**e**, **f**) 80 vol.% acetone. Red lines show single-exponential fit (Equation S1), green lines show bi-exponential fit (Equation S2).

T_2 relaxation curves with single-exponential decay were fitted with equation

$$M(t) = M_0 \exp\left(-\frac{T_2}{t}\right) + y_0 \tag{S1}$$

where T_2 is spin-spin relaxation time, M_0 is pre-exponential factor and y_0 is constant.

T_2 relaxation curves with bi-exponential decay were fitted with equation

$$M(t) = M_0^1 \exp\left(-\frac{T_2^1}{t}\right) + M_0^2 \exp\left(-\frac{T_2^2}{t}\right) + y_0 \tag{S2}$$

where T_2^1 and T_2^2 are components of spin-spin relaxation time, M_0^1 and M_0^2 are pre-exponential factors and y_0 is constant.
